# Health state utilities associated with treatment process for oral and injectable GLP-1 receptor agonists for type 2 diabetes

**DOI:** 10.1007/s11136-021-02808-2

**Published:** 2021-04-22

**Authors:** Louis S. Matza, Katelyn N. Cutts, Katie D. Stewart, Kirsi Norrbacka, Luis-Emilio García-Pérez, Kristina S. Boye

**Affiliations:** 1 Patient-Centered Research, Evidera, 7101 Wisconsin Avenue, Suite 1400, Bethesda, MD 20814 USA; 2 Eli Lilly Finland, Helsinki, Finland; 3 Lilly S.A, Alcobendas, Spain; 4 Eli Lilly and Company, Indianapolis, IN USA

**Keywords:** (4–6): Health state utility, Treatment process utility, Utility, Route of administration, Type 2 diabetes, Glucagon-like peptide-1 receptor agonist, GLP-1 RA

## Abstract

**Purpose:**

Previous research suggests that treatment process can have an influence on patient preference and health state utilities. This study examined preferences and estimated utilities for treatment processes of two daily oral treatment regimens and two weekly injectable regimens for treatment of type 2 diabetes (T2D).

**Methods:**

Participants with T2D in the UK reported preferences and valued four health state vignettes in time trade-off utility interviews. The vignettes had identical descriptions of T2D but differed in treatment process: (1) daily simple oral treatment (tablets without administration requirements), (2) daily oral semaglutide (with administration requirements per product label), (3) weekly dulaglutide injection, (4) weekly semaglutide injection.

**Results:**

Interviews were completed by 201 participants (52.7% male; mean age = 58.7). Preferences between treatment processes varied widely. Mean utilities were 0.890 for simple oral, 0.880 for oral semaglutide, 0.878 for dulaglutide injection, and 0.859 for semaglutide injection (with higher scores indicating greater preference). All pairwise comparisons found statistically significant differences between utilities (p < 0.01), except the comparison between oral semaglutide and the dulaglutide injection (p = 0.49).

**Conclusions:**

Results suggest that routes of administration cannot be compared using only the simplest descriptions (e.g., oral versus injectable). Dose frequency and specific details of the treatment process administration had an impact on patient preference and health state utilities. The utilities estimated in this study may be useful in cost-utility models comparing these treatments for T2D. Results also suggest that it may be helpful to consider patient preferences for treatment process when selecting medications for patients in clinical settings.

**Supplementary Information:**

The online version contains supplementary material available at 10.1007/s11136-021-02808-2.

## Introduction

A growing body of literature suggests the process of receiving treatment can have an impact on quality of life and health state utilities, which are values representing the strength of preference for various health states [1–4]. These “treatment process utilities” can be used as inputs in cost-utility analyses (CUAs), which are conducted to inform decisions regarding healthcare resource allocation [5]. The impact on utility has been estimated for a wide range of treatment process attributes, including route of administration (ROA), dose frequency, dose flexibility, and injection device characteristics [1, 6]. Small differences in utility associated with treatment process can influence the outcomes of a CUA and subsequent decision-making based on the model results. Treatment process utilities can also provide insight into patient preference, which has been highlighted as a “major factor driving the choice of medication” for patients with type 2 diabetes (T2D) in a recently published consensus report by the American Diabetes Association and the European Association for the Study of Diabetes [7]. This consensus report emphasizes that patient preference is influenced by treatment attributes beyond efficacy and safety, including ROA.

ROA is often a primary focus of research on treatment process utilities. A range of published studies have estimated utility differences between oral and injectable treatment, and the oral ROA tends to be preferred as indicated by higher utility scores [8–10]. Because studies estimating process utility usually use vignette-based methods [1], the utility differences between oral and injectable ROA are mostly based on valuations of health state descriptions that vary with regard to ROA.

Several vignette-based studies have been conducted to estimate utilities associated with treatment process of glucagon-like peptide-1 (GLP-1) receptor agonists for T2D [6, 9, 11, 12]. Until recently, all medications in this class were injectable, and therefore, utility studies have focused on aspects of the injection process, such as injection frequency, injection preparation requirements, and the injection devices [6, 9, 11, 12]. These studies have usually included a comparator health state describing oral treatment, which has consistently been preferred over the regimens including both oral and injectable treatment [6, 9, 11]. However, the health state vignettes describing oral treatment regimens have not included specific details. For example, a typical description of oral treatment in these studies is “You take an oral medication (tablet) every day” [6, 9, 11].

The first oral formulation of a GLP-1 receptor agonist, semaglutide, has recently been approved in several countries [13–15]. Unlike some oral T2D medications such as metformin with relatively simple treatment administration instructions [16], oral semaglutide requires the patient to take specific steps for the product to be effective. For example, the package insert instructs patients to take the medication on an empty stomach and avoid eating, drinking, or taking other oral medications for 30 min after taking the medication [14, 15]. Because previously published utilities for the oral ROA were derived from vignettes without these requirements [6, 9, 11], these published utilities may not be applicable to oral semaglutide.

The purpose of this study was to examine patient preferences and estimate health state utilities associated with the treatment process of GLP-1 receptor agonists for treatment of T2D, including oral semaglutide and two weekly injectable treatments, dulaglutide [17] and semaglutide [18]. As a comparator, respondents also valued a “simple oral” health state that has been used in previous studies. In contrast to previous studies in which injectable GLP-1 receptor agonists have been presented in combination with oral treatment [6, 9, 11], this is the first study to elicit preferences and utilities for the oral and injectable treatment processes as monotherapy. With this approach, this study was designed to provide insight into utility differences between oral and injectable ROA, while highlighting the impact of treatment administration details on patient preference.

## Methods

### Overview of study design

This study was designed to elicit preferences and estimate health state utilities associated with the treatment process of GLP-1 receptor agonists for treatment of T2D. Several health technology assessment (HTA) guidelines recommend that utilities are derived from generic preference-based measures when possible [19–22]. However, because generic instruments such as the EQ-5D are designed to assess overall health status, they are unlikely to be sensitive to differences in treatment process attributes such as mode of administration. In contrast, vignette-based methods are well-suited for isolating the utility impact of treatment process attributes such as mode of administration. Therefore, like most studies designed to estimate treatment process utilities [1], the current study used the vignette approach.

Four health state vignettes (also called scenarios or health states) were developed based on previous literature and package inserts for the relevant medications (see Online Appendix A for complete text of these health states). The health states each began with the same description of T2D, followed by a description of the treatment process, which varied across the four health states. Because all aspects of the four health states were identical except for treatment process, any difference in preference or utility can be attributed entirely to the treatment process. Two oral and two injectable health states were included so that preferences could be examined not only between oral and injectable ROA, but also between different oral processes and between different injectable processes.

The health states were used in a utility valuation study with a sample of participants with T2D in two UK locations (Edinburgh, London) in December 2019 and January 2020. Preferences between the four treatment processes were assessed by asking participants to rank the health states from most preferable to least preferable. Then, utilities were elicited in a time trade-off task (TTO). Written informed consent was obtained from each participant, and the study protocol was approved by an independent institutional review board (Ethical & Independent Review Services; Study 19,038-01).

### Health state vignettes

The four health states began with the same description of T2D, which was derived from previous studies using similar methods [6, 9, 11, 23]. This series of bullet points referred to blood sugar levels, typical symptoms, and body weight. The description of T2D was intended to be general so that it could apply to many patients with T2D and plausibly be paired with any of the treatment process descriptions that followed in each of the four health states. The purpose of this T2D description was to provide brief context for the description of treatment process that followed. Most importantly, this description of diabetes remained constant so that it did not have an impact on preference between the four health states.

After the description of T2D, each health state continued by describing the treatment administration process, which varied across the four health states. Health state A described a simple daily oral medication administration without other specific requirements (“You take oral medication [pills or tablets] every day”). This description has been used in previously published studies [6, 9, 11, 23].

Health state B described administration of oral medication with requirements from the US package insert for oral semaglutide [15]. These bullet point statements were adopted directly from the language in this package insert, and exact language from the label was used as much as possible. Requirements included taking the tablet “on an empty stomach when you first wake up,” “with a sip of plain water,” and “wait at least 30 minutes after taking this tablet before eating, drinking, or taking other oral medications.” The US label was used because it was available at the time this study was conducted. The European Union (EU) label [14] was released after data collection was complete. The medication administration requirements in the US and EU labels are almost identical, with only one minor difference relevant to this health state. Both the US and EU versions direct patients to take the medication on an empty stomach and wait 30 min before eating or drinking, but they differ in their wording. The US version says the medication should be taken “when you first wake up.” The EU version says medication may be taken “at any time of day” but then instructs users to “wait at least 30 minutes before having your first meal or drink of the day or taking other oral medicines.” Despite this difference in wording, the meaning of these instructions appears to be the same.

Health states C and D described injectable treatment administration processes for two commonly used GLP-1 receptor agonists, dulaglutide (C) and semaglutide (D). While both medications are in the same class and administered weekly, they differ with regard to the injection device and steps for preparing the device. The health states described the weekly dose frequency, injection pen, steps for preparing the pen, and injection procedures. Dulaglutide was selected as a comparator because it is one of the most commonly used GLP-1 receptor agonists across multiple countries [24] and because its treatment process attributes have been preferred over procedures for administering other GLP-1 receptor agonists in previous studies [6, 11, 25]. Dulaglutide is administered with a single-dose, single-use auto-injector pen that does not require handling the needle [26]. Injectable semaglutide was selected as the other comparator because it has a different treatment process involving a multi-use, pre-filled injection pen that requires needle attachment/disposal and dose dialing with each use [27].

Health states C and D were based on the EU Instructions for Use (IFU) provided as a package insert with dulaglutide [17] and injectable semaglutide [18], and exact language from the IFUs was used as much as possible. Both health states were included in a previously published utility elicitation study [11], but one revision was made for the current study. In the previous study, both health states described the weekly injection as an addition to daily oral medication. For the current study, the weekly injection was presented as monotherapy.

### Participants

Participants were recruited through newspaper advertisements, online advertisements, and distribution of fliers. Potential participants responded via email or voicemail to provide their contact information. They were screened using a standardized screening script to confirm eligibility. All participants were required to be between 30 and 75 years old, reside in the UK, and have been diagnosed with T2D. Participants who received medication for diabetes were required to provide proof of treatment (e.g., a prescription for medication used to treat T2D). Participants who had been treated with either dulaglutide or semaglutide were not eligible. Although this sample cannot be considered nationally representative, recruitment targets were set based on the UK census [28] and the UK T2D population [29] to ensure that no demographic groups were over-represented with regard to age, gender, ethnic/racial background, and unemployment status.

The recruitment advertisements described the study as a “type 2 diabetes survey study” with an “interview about hypothetical health choices.” No further details about the study content were provided in the advertisement or the standardized screening script. For example, patients were not told in advance that they would be asked to consider oral and injectable treatment processes. Therefore, there was no apparent selection bias that would have contributed to preferences for oral or injectable treatments.

### Pilot study

A pilot study was conducted with 41 participants in London with T2D (53.7% male; mean age = 55.2 years) to assess the clarity and comprehensibility of the interview procedures and health states. Participants completed the TTO valuation and then provided feedback. Health states were revised based on participant feedback and interviewer observations to ensure that health states were clear and easy to understand. The final versions of the health states and methods were used with the last 30 pilot study participants. These participants consistently reported that the health states and procedures were clear and comprehensible. These participants were not included in the main valuation analysis sample.

### Utility interview procedures and scoring

A utility elicitation study was conducted to value the four health states finalized in the pilot study. Six trained interviewers followed a semi-structured interview guide to conduct one-on-one interviews in private rooms. Each interviewer was observed by the study principal investigator multiple times to ensure consistency in interview procedures.

Participants were introduced to the health states, which were each presented on individual cards. After each participant reported that they understood the health states and had no further questions about health state content, they were asked to rank the health states from most preferable to least preferable. Respondents were also asked to explain reasons for their preferences between the health states.

After completing the ranking, participants valued the health states in a TTO task with a 20-year time horizon. For each health state, participants were offered a choice between spending a 20-year period in the health state versus spending varying amounts of time in full health. Choices were presented in one-year (i.e., 5%) increments, alternating between longer periods of time and shorter periods of time (i.e., 20 years, 0 years [dead], 19 years, 1 year, 18 years, 2 years, 17 years, 3 years…). Utility (*u*) was assigned based on the point of indifference between years in the health state being valued (*y*) and years in full health (*x*) (followed by dead). Utility with the anchors of dead (0) and full health (1) was then calculated as *u* = *x/y*.

When a respondent perceived a health state to be worse than dead, the interviewer adjusted the TTO task as described in previous literature [30]. Participants were offered a choice between dead (choice 1) and a 20-year life span (choice 2) beginning with varying amounts of time in the health state being rated, followed by full health for the remainder of the 20 years. The resulting negative utility scores were calculated with a bounded scoring approach that has been used to avoid highly skewed distributions for negative utility scores (*u* = *-x/y*, where *x* is time in full health, and *y* is the total life span of choice 2).

### EQ-5D-5L

The EQ-5D-5L was administered to characterize the sample. The EQ-5D-5L is a self-administered, generic, preference-weighted measure designed to assess health status [31]. The instrument includes five dimensions assessing mobility, self-care, usual activities, pain/discomfort, and anxiety/depression. For each dimension, the respondent selects one of five response options, and these responses were used to obtain an index score using a published mapping algorithm [32].

### Statistical analysis procedures

Statistical analyses were completed with SAS (version 9.4). Continuous variables (including utilities and utility difference scores) were summarized as means and standard deviations, and categorical variables were presented as frequencies and percentages. Paired t-tests were conducted to test whether there were statistically significant pairwise differences between heath state utilities (e.g., the utility of health state A vs. the utility of health state B). Paired t-tests were conducted to test for subgroup differences in utility scores. For these analyses, participants were categorized based on age, gender, employment status, education level, geographic location, and current treatment.

## Results

### Sample characteristics

A total of 227 potential participants were scheduled for interviews, and 207 attended their interviews. Two of the 207 participants had difficulty understanding the utility interview procedures and were therefore unable to provide valid data. Two individuals declined to sign the consent form and did not complete any study procedures. An additional two individuals were found to be ineligible during review of inclusion criteria at the beginning of the interview (one had previously used dulaglutide or semaglutide; the other had not received a diagnosis of T2D). Therefore, the analysis includes data from 201 interviews (88 Edinburgh, 113 London).

The sample was 52.7% male, with a mean age of 58.7 years. The majority of participants reported ethnicity as White (80.1%). More than half of the participants were employed (33.8% full-time and 22.9% part-time), while less than half (42.8%) reported having a university degree. There was no significant difference between the London and Edinburgh samples in age, gender, employment status, or marital status. Compared with the London sample, the Edinburgh sample had a greater percentage of White participants (92.0% vs.70.8%) and fewer participants with a university degree (31.8% vs. 51.3%). Demographic information is presented in Table 1.

**Table 1:** Demographic and clinical characteristics (N = 201)

Characteristics	Total Sample (N = 201)	Edinburgh (N = 88)	London (N = 113)	P-value^a^
Age (Mean, SD)	58.7 (8.3)	58.8 (8.6)	58.7 (8.1)	0.8907
Male (n, %)	106 (52.7%)	47 (53.4%)	59 (52.2%)	0.8661
Ethnicity (n, %)				
White	161 (80.1%)	81 (92.0%)	80 (70.8%)	0.0041
African, Caribbean, or Black	10 (5.0%)	3 (3.4%)	7 (6.2%)	
Asian	17 (8.5%)	3 (3.4%)	14 (12.4%)	
Mixed ethnicity^b^	9 (4.5%)	1 (1.1%)	8 (7.1%)	
Other^c^	4 (2.0%)	0 (0.0%)	4 (3.5%)	
Marital Status (n, %)				
Single	103 (51.2%)	39 (44.3%)	64 (56.6%)	0.0830
Married/Cohabitating/Living with partner	98 (48.8%)	49 (55.7%)	49 (43.4%)	
Employment Status (n, %)				
Full-time work	68 (33.8%)	25 (28.4%)	43 (38.1%)	0.0760
Part-time work	46 (22.9%)	17 (19.3%)	29 (25.7%)	
Other^d^	87 (43.3%)	46 (52.3%)	41 (36.3%)	
Education Level (n, %)				
University degree	86 (42.8%)	28 (31.8%)	58 (51.3%)	0.0055
No University degree	115 (57.2%)	60 (68.2%)	55 (48.7%)	
Diabetes Medication (n, %)				
No medication treatment	33 (16.4%)	22 (25.0%)	11 (9.7%)	0.0026
Oral medication	139 (69.2%)	58 (65.9%)	81 (71.7%)	
Insulin	6 (3.0%)	4 (4.5%)	2 (1.8%)	
Oral and non-insulin injectable	5 (2.5%)	2 (2.3%)	3 (2.7%)	
Oral and insulin	18 (9.0%)	2 (2.3%)	16 (14.2%)	

^a^P values are based on comparisons between London and Edinburgh subgroups, using t-tests for continuous variables and Chi-square analyses for categorical variables

^b^Mixed ethnicity includes: Asian and European (n = 1), Asian and Scottish Muslim (n = 1), Black and British (n = 1), English/Singaporean (n = 1), Iranian (n = 1), Irish and Mauritian (n = 1), Mixed Caribbean/Irish/English (n = 1), prefer not to say (n = 1), and Welsh and South African (n = 1)

^c^Free-text responses provided by participants include: Maltese (n = 1), Latin-American (n = 1), oriental/Indonesia (n = 1), and prefer not to say (n = 1)

^d^Other work includes: Retired (n = 61), disabled (n = 15), homemaker/housewife (n = 4), unemployed (n = 4), student (n = 1), charity worker (n = 1), and doing voluntary work (n = 1)

Participants reported being diagnosed with T2D an average of 8.3 years (SD = 7.0) prior to their interview. Most participants were currently receiving medication to treat their diabetes (83.6%) (Table 1). The most commonly reported health conditions included: hypertension (28.9%), anxiety (23.4%), depression (20.4%), and arthritis (19.4%). The mean EQ-5D-5L index score was 0.81 (SD = 0.23), which is similar to scores for patients with T2D without serious diabetes-related complications in previous studies [33].

### Health state rankings and preferences

Participants ranked the four health states in order of preference from 1 (most preferable health state) to 4 (least preferable health state) (Table 2). The simple oral health state (A) was ranked as most preferable by a majority of participants (n = 139; 69.2%), while 60 participants (29.9%) said one of the injection health states (C or D) was most preferable. The semaglutide injection health state (D) was most commonly ranked as least preferable (n = 144; 71.6%).

**Table 2:** Health state rankings^a^ (N = 201)

Health states varying by treatment process (n, %)	1 = Most preferred	2	3	4 = Least preferred
A. Simple Oral	139 (69.2%)	30 (14.9%)	30 (14.9%)	2 (1.0%)
B. Semaglutide Oral	2 (1.0%)	102 (50.7%)	53 (26.4%)	44 (21.9%)
C. Dulaglutide Injection	49 (24.4%)	41 (20.4%)	100 (49.8%)	11 (5.5%)
D. Semaglutide Injection	11 (5.5%)	28 (13.9%)	18 (9.0%)	144 (71.6%)

^a^Rankings had a possible range from 1 to 4, with lower numbers indicating more preferred health states

Ranking for each individual health state relative to each other health state is presented in Table 3. For example, almost all the participants (97.0%) preferred the simple oral health state (A) over the oral semaglutide health state (B), and 88.6% preferred the dulaglutide health state (C) over the injectable semaglutide health state (D). Preferences between the oral semaglutide (B) and injectable dulaglutide (C) health states varied. While 53.2% of the sample preferred the daily oral semaglutide health state, 46.8% preferred weekly injectable dulaglutide. Participants provided reasons for their preferences, and a selection of these qualitative responses is provided in Table 4.

**Table 3:** Preferences between pairs of health states (N = 201)

Pairs of health states differing in treatment process	Respondents preferring each health state N (%)
(A) Prefer Simple Oral	195 (97.0%)
(B) Prefer Oral Semaglutide	6 (3.0%)
(A) Prefer Simple Oral	144 (71.6%)
(C) Prefer Dulaglutide Injection	57 (28.4%)
(A) Prefer Simple Oral	168 (83.6%)
(D) Prefer Semaglutide Injection	33 (16.4%)
(B) Prefer Oral Semaglutide	107 (53.2%)
(C) Prefer Dulaglutide Injection	94 (46.8%)
(B) Prefer Oral Semaglutide	150 (74.6%)
(D) Prefer Semaglutide Injection	51 (25.4%)
(C) Prefer Dulaglutide Injection	178 (88.6%)
(D) Prefer Semaglutide Injection	23 (11.4%)

**Table 4:** Quotations from participants explaining their preferences between the four health states

Preferences	Selected quotations
Participants who preferred oral over injectable route of administration	“I could never inject myself. I would have to get a GP to do it for me. No way I’d inject myself.”
	“I would rather have the simpleness of taking tablets.”
	“I would rather take a tablet than inject. I’m fed up with injecting.”
	“Easier to do…less stigma compared to injections.”
	“I don’t really like injections. I’m scared of needles.”
Participants who preferred one of the weekly injectable health states over the daily oral semaglutide health state because of dose frequency	“Comfortable, easy, straight forward, and you only have to do it once a week.” (referring to dulaglutide injection)
	“Taking an injection once a week reduces the stress of taking medicine once a day. I sometimes miss my daily doses.”
	“I’m not usually a fan of needles, but this one seems so easy, it’s only once a week and you don’t see the needle.”
	“The weekly one is more convenient.”
	“The simpler, the better. Once a week is key.”
Participants who preferred one of the weekly injectable health states over the daily oral semaglutide health state because of the simplicity of the injection device	“This seems a lot easier a system to me.” (referring to dulaglutide injection over oral semaglutide and injectable semaglutide)
	“[The dulaglutide injection] seems more straightforward, everything is done for you, you don’t have to worry about having an empty stomach.”
	“[The dulaglutide injection] is the quickest and easiest if you are a busy person. It’s all down to convenience, quickness, and easiness.”
	“[The dulaglutide injection] appears to be easy and non-invasive. The work is done for you. It seems very straightforward.”
	“[The dulaglutide injection] is the easiest. Dosage is fixed, you don’t see the needle, you only do it once a week so you don’t have to worry about it.”
Participants who preferred one of the weekly injectable health states over the daily oral semaglutide health state due to requirements of the oral semaglutide administration procedure	“I don’t like the idea of having to wait to eat.” (referring to oral semaglutide)
	“It’s just going to start affecting your quality of life to have the eating restrictions.”
	“[Oral semaglutide] would be the least preferable because I’m a nightshift worker. If I’ve eaten through the night and have fluids, this would be a problem.”
	“You have to wait 30 minutes to eat and you have to take it on an empty stomach. I like to have coffee first thing when I wake up. I would mess it up every day.”
	“[Oral semaglutide] is too much faffing about.”

### Health state utilities

**Fig. 1:**
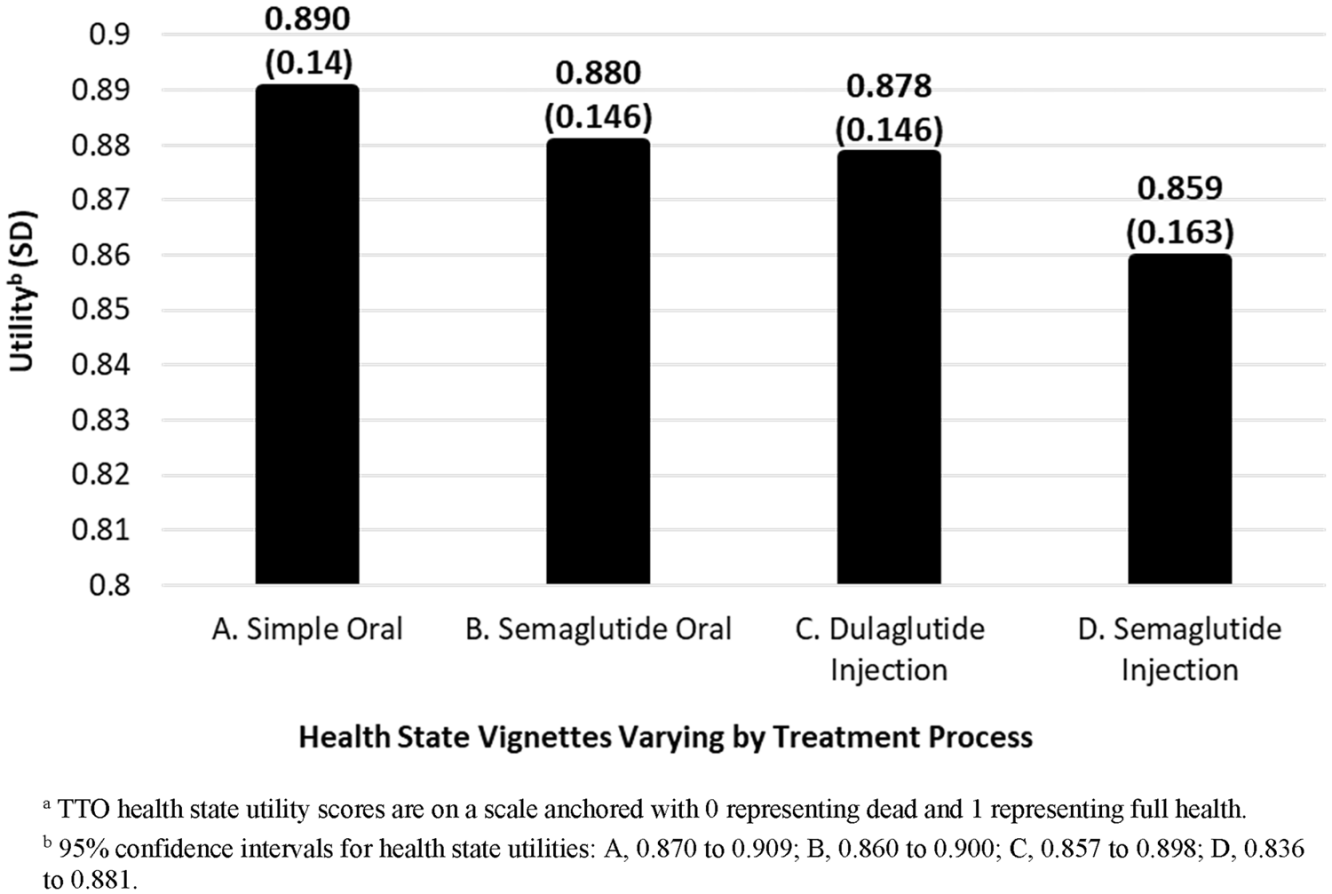
Health state utility scores^a^ (N = 201)

The simple oral health state had the highest mean utility score (0.890), followed by semaglutide oral (0.880), dulaglutide injection (0.878), and semaglutide injection (0.859) (Fig. 1). Pairwise comparisons between health state utilities were performed with t-tests (Table 5). There was no significant difference between utilities of the oral semaglutide health state (B) and the dulaglutide health state (C). All other pairwise comparisons found statistically significant differences between utilities (p < 0.001).

**Table 5:** T-tests comparing health state utilities^a^ (N = 201)

Pairs of health states	Mean health (SD) state utility	Mean (SD) difference score	T value (paired)	Adjusted p-value^b^
A. Simple Oral	0.890 (0.140)	0.010 (0.033)	4.2	.0002
B. Semaglutide Oral	0.880 (0.146)			
A. Simple Oral	0.890 (0.140)	0.012 (0.041)	4.2	.0002
C. Dulaglutide Injection	0.878 (0.146)			
A. Simple Oral	0.890 (0.140)	0.031 (0.084)	5.2	.0001
D. Semaglutide Injection	0.859 (0.163)			
B. Semaglutide Oral	0.880 (0.146)	0.002 (0.051)	0.7	.49
C. Dulaglutide Injection	0.878 (0.146)			
B. Semaglutide Oral	0.880 (0.146)	0.021 (0.082)	3.7	.0009
D. Semaglutide Injection	0.859 (0.163)			
C. Dulaglutide Injection	0.878 (0.146)	0.019 (0.075)	3.5	0.001
D. Semaglutide Injection	0.859 (0.163)			

^a^TTO scores are on a scale anchored with 0 representing dead and 1 representing full health

^b^P values for these t-tests were adjusted for multiple comparisons using the Bonferroni step-down procedure [47]

Utilities were compared between subgroups. For health states A, B, and C, there were no statistically significant between-group differences with regard to age (median split: younger [n = 108] vs. older [n = 93]), gender, employment status (retired [n = 61] vs. employed full-time or part-time [n = 114]), geographic location (Edinburgh [n = 88] vs. London [n = 113]), education level (university degree [n = 86] vs. no university degree [n = 115]), or current treatment (three groups: only oral medication [n = 139]; injectable medication with or without oral medication [n = 29]; no medication [n = 33]). The fourth health state (D representing injectable semaglutide) had the lowest mean utility across all subgroups, and there was no significant difference by geographic location, education level, treatment group, or employment status. However, for health state D, younger participants had a significantly greater mean utility than older participants (0.883 vs. 0.831; p < 0.05), and men had a significantly greater mean utility than women (0.882 vs. 0.833; p < 0.05).

Only one participant had negative utility scores, indicating that she perceived health states B, C, and D to be worse than dead. All other participants had positive utilities for all four health states.

## Discussion

In previous studies examining patient preference between oral and injectable ROAs, results have tended to favor oral over injectable treatment [34–41]. These preferences have frequently led to greater health state utilities for oral treatment than for injectable treatment [8, 10, 42, 43]. However, current results suggest that preference for oral over injectable treatment may not be as consistent or straightforward as previously reported. When considering detailed descriptions of treatment administration, almost half (46.8%) of the participants preferred the dulaglutide injection over the oral semaglutide regimen, and the utility difference between these two health states was not statistically significant. This suggests that studies assessing preference and utilities associated with treatment process should consider not only ROA, but also details such as dose frequency and complexity of treatment administration.

Patients’ explanations of their preferences between oral and injectable ROAs illustrate the substantial variation in patient perceptions and priorities (Table 4). Some patients reported that they would always prefer oral over injectable treatment. Patients who preferred one or both injectable options over oral semaglutide mentioned inconveniences of the oral semaglutide administration requirements, the convenience of a weekly injection rather than daily oral treatment, and/or the simplicity of the dulaglutide injection device. These qualitative responses show that specific characteristics of the treatment process can have an impact on preference for ROA. It may be useful to consider patient preference for treatment process characteristics when selecting treatments for individual patients in clinical settings.

The current study design also allows for differentiation between two variations of oral administration and between two types of injectable administration. Almost all respondents (97.0%) preferred the simple oral health state (A) over the oral semaglutide health state (B), and the difference in utility between health states A and B was statistically significant. This suggests that previously published utilities representing simple oral treatment [6, 9, 11, 23] should not be used to represent oral regimens that have unique administration requirements, such as oral semaglutide. Study results also highlight patient preference for simpler injection devices, which has been demonstrated in previous research. The percentage of patients preferring the dulaglutide device over the semaglutide injection device (88.6%) was almost identical to results of a previous study examining this preference in Italy (88.4%) [11].

These findings have implications for research on treatment process utilities. Studies designed to estimate treatment process utilities most frequently use vignette-based methods because, unlike generic instruments, these methods can isolate the impact of treatment process on utility [1]. To ensure that utilities accurately represent the impact of treatment process on preference and quality of life, vignettes should include not only the ROA, but also specific characteristics of each ROA that could be important to patients. In addition, when incorporating the resulting utilities in a CUA, modelers should carefully consider whether the health state vignettes accurately represent the treatments being compared in the economic model. Because details of the treatment process can have an impact on the resulting utility, a utility for one treatment process may not be applicable to another treatment with the same ROA but different treatment administration requirements. To facilitate decisions regarding appropriateness of utilities for subsequent models, publications of vignette-based studies should present the full language of each vignette so that modelers and HTA reviewers can make an informed decision about whether the utilities are truly applicable in each specific situation.

Results of the current study may be useful in economic models examining and comparing the value of oral treatment without administration requirements, oral semaglutide, dulaglutide, and injectable semaglutide for treatment of T2D. The utility difference scores presented in Table 5 may be used to adjust utility values estimated for specific treatment groups using a standardized instrument like the EQ-5D. For example, if a model were comparing injectable semaglutide to dulaglutide, EQ-5D values from a clinical trial may not reflect differences in quality of life associated with ROA. Therefore, modelers could adjust the value of the dulaglutide health state upward by 0.019 (i.e., difference between utilities of health states C and D in Table 5) using an additive approach. The adjustment could also be performed with a multiplicative approach by multiplying utilities in Fig. 1 by the utilities from a generic instrument completed in a trial [44, 45]. This type of treatment process adjustment may be included in a base case analysis or a sensitivity analysis following the initial CUA to provide additional information regarding the potential value of the treatments.

Several methodological limitations should be considered when interpreting results from this study. First, an inherent limitation of vignette-based methods is that the utility scores are based on preference for health state descriptions rather than real-world experience of the treatments. The extent to which the resulting utilities may differ from preferences of patients with firsthand experience of each treatment is unknown. It is possible that after extended exposure, a patient could become comfortable with treatment processes that initially seemed aversive.

Second, because these vignettes were designed to isolate the utility impact of treatment process, it was necessary to hold other aspects of the health states constant (e.g., treatment efficacy, treatment-related adverse events). Therefore, the results reflect preferences associated with treatment process, but not other aspects of these treatments for T2D. When comparing among treatments in economic modeling or in clinical settings, treatment process must be considered in the context of other factors such as efficacy and safety. While the current study highlights potentially important differences in treatment process, results do not provide insight into the utility impact of these broader aspects of treatment.

Third, although patients can clearly express and explain preferences among treatment process options, the magnitude of the utility differences was small compared to utility differences typically associated with clinical outcomes such as treatment response, symptom reduction, and adverse events. The statistically significant differences in Table 5 ranged from 0.010 to 0.031. Utility differences of less than 0.05 are typical when examining differences between ROAs [6, 9–11, 42, 43, 46]. While treatment process tends to have less impact on utilities than clinical outcomes, these small utility differences can have an impact on the outcomes of a CUA. Furthermore, these treatment process utilities can help ensure that patient preferences among treatments are considered as a factor contributing to cost-effectiveness.

A fourth limitation stems from the sample characteristics. Some HTA agencies prefer that utilities are based on general population values [20–22]. However, the current study was conducted with a patient sample, rather than a general population sample. The advantage of the patient sample is that the study results yield not only utilities, but also patient preferences for ROA, which could be important to consider in clinical settings. The extent to which utilities from the current study may differ from general population values is not known.

Future research could build on current findings by examining subgroup differences in preferences for treatment process among people with T2D. For health states A, B, and C, there were no subgroup differences in utility. However, with health state D, two subgroup differences emerged. For most participants, D was the least preferred health state, describing a treatment process that was perceived as relatively complex. On average, older participants and women in this sample were more averse to this injectable treatment process than younger participants or men. However, this is the first study to find these subgroup differences, and these findings should be interpreted with caution until they can be replicated in future research.

Overall, this study adds to research on patient preference and health state utilities associated with treatment process. Findings suggest that ROAs cannot be compared using only the simplest descriptions, such as “oral vs. injectable.” Dose frequency and the details of treatment administration can have an impact on patient preference, and therefore, they should be considered when examining patient preference, estimating treatment process utilities, using process utilities in economic modeling, and selecting treatments for patients in clinical settings.

## Supplementary Information

Below is the link to the electronic supplementary material.

Supplementary file1 (DOCX 17 KB)

## Data Availability

Data can be made available upon reasonable request.

## References

[1] Brennan VK, Dixon S (2013). Incorporating process utility into quality adjusted life years: a systematic review of empirical studies. PharmacoEconomics.

[2] De Abreu LR, Haas M, Hall J, Viney R (2017). Valuing meta-health effects for use in economic evaluations to inform reimbursement decisions: a review of the evidence. PharmacoEconomics.

[3] Higgins A, Barnett J, Meads C, Singh J, Longworth L (2014). Does convenience matter in health care delivery? A systematic review of convenience-based aspects of process utility. Value in Health.

[4] Stewart KD, Johnston JA, Matza LS, Curtis SE, Havel HA, Sweetana SA (2016). Preference for pharmaceutical formulation and treatment process attributes. Patient Preference and Adherence.

[5] Brazier J, Ratcliffe J, Salomon JA, Tsuchiya A (2017). Measuring and Valuing Health Benefits for Economic Evaluation.

[6] Matza LS, Boye KS, Stewart KD, Davies EW, Paczkowski R (2017). Health state utilities associated with attributes of weekly injection devices for treatment of type 2 diabetes. BMC Health Services Research.

[7] Davies MJ, D'Alessio DA, Fradkin J, Kernan WN, Mathieu C, Mingrone G (2018). Management of hyperglycaemia in type 2 diabetes, 2018. A consensus report by the American Diabetes Association (ADA) and the European Association for the Study of Diabetes (EASD). Diabetologia.

[8] Holko P, Kawalec P, Mossakowska M (2018). Quality of life related to oral, subcutaneous, and intravenous biologic treatment of inflammatory bowel disease: a time trade-off study. European Journal of Gastroenterology & Hepatology.

[9] Matza LS, Boye KS, Jordan JB, Norrbacka K, Gentilella R, Tiebout AR (2018). Patient preferences in Italy: health state utilities associated with attributes of weekly injection devices for treatment of type 2 diabetes. Patient Preference and Adherence.

[10] Mittmann N, Craven BC, Gordon M, MacMillan DH, Hassouna M, Raynard W (2005). Erectile dysfunction in spinal cord injury: a cost-utility analysis. Journal of Rehabilitation Medicine.

[11] Boye KS, Matza LS, Stewart KD, Jordan J, Biricolti G, Del Santo S (2019). Patient preferences and health state utilities associated with dulaglutide and semaglutide injection devices among patients with type 2 diabetes in Italy. Journal of Medical Economics.

[12] Polster M, Zanutto E, McDonald S, Conner C, Hammer M (2010). A comparison of preferences for two GLP-1 products–liraglutide and exenatide–for the treatment of type 2 diabetes. Journal of Medical Economics.

[13] Hedrington MS, Davis SN (2019). Oral semaglutide for the treatment of type 2 diabetes. Expert Opinion on Pharmacotherapy.

[14] Novo Nordisk A/S. EU Summary of Product Characteristics: Rybelsus 3 mg, 7 mg, and 14 mg tablets. Denmark; April 2020.

[15] Novo Nordisk A/S. Highlights of Prescribing Information - RYBELSUS (semaglutide) tablets 7 mg / 14 mg, for oral use (US label). Plainsboro, NJ; Initial Approval: 2017. Revised: September 2019.

[16] Bristol-Myers Squibb. Full prescribing information: Glucophage (metformin hydrochloride) tablets, for oral use and glucophage XR (metformin hydrochloride) extended-release tablets, for oral use Princeton, NJ; Initial US Approval: 1995; Revised: May 2018.

[17] Eli Lilly and Company. Product Information for Trulicity 0.75mg and 1.5mg solution for injection in pre-filled pen. The Netherlands; 21 Nov 2014.

[18] Novo Nordisk A/S. Product Information for Ozempic 0.25mg, 05mg, and 1mg solution for injection in pre-filled pen. Denmark; 08 February 2018.

[19] Haute Autorité de santé (HAS). A Methodological Guide: Choices in Methods for Economic Evaluation. In: Department of Economics and Public Health Assessment, editor. Saint-Denis La Plaine, France; October 2012. p. 55

[20] Canadian Agency for Drugs and Technologies in Health (CADTH). Guidelines for the Economic Evaluation of Health Technologies: Canada. 4th ed. Ottawa, CAN; March 2017. p. 76

[21] National Institute for Health and Care Excellence (NICE). Guide to the methods of technology appraisal 2013. London, UK; 4 April 2013. p. 93. 27905712

[22] Pharmaceutical Benefits Advisory Committee (PBAC). Guidelines for preparing a submission to the Pharmaceutical Benefits Advisory Committee. In: Australian Government DoH, editor. Canberra, ACT; September 2016. p. 216

[23] Boye KS, Matza LS, Walter KN, Van Brunt K, Palsgrove AC, Tynan A (2011). Utilities and disutilities for attributes of injectable treatments for type 2 diabetes. The European Journal of Health Economics.

[24] Divino V, Boye KS, Lebrec J, DeKoven M, Norrbacka K (2019). GLP-1 RA Treatment and Dosing Patterns Among Type 2 Diabetes Patients in Six Countries: A Retrospective Analysis of Pharmacy Claims Data. Diabetes Therapy.

[25] Matza LS, Boye KS, Stewart KD, Coyne KS, Wullenweber PK, Cutts KN (2020). Assessing patient PREFERence between the dulaglutide pen and the semaglutide pen: A crossover study (PREFER). Diabetes, Obesity & Metabolism.

[26] Eli Lilly and Company. Instructions for Use: TRULICITY® (Trū-li-si-tee) (dulaglutide) injection, for subcutaneous use - 0.75 mg/0.5 mL Single-Dose Pen once weekly. Indianapolis, IN; 2017.

[27] Novo Nordisk A/S. Instructions for Use: OZEMPIC® (semaglutide) injection, for subcutaneous use - 0.5 mg/1 mg. 2017.

[28] Office for National Statistics (ONS). 2011 Census: Population and Household Estimates for the United Kingdom. Table 3 2011 Census: Usual resident population by five-year age group and sex, local authorities in the United Kingdom. Last Updated: 17 December 2012.

[29] Zghebi SS, Steinke DT, Carr MJ, Rutter MK, Emsley RA, Ashcroft DM (2017). Examining trends in type 2 diabetes incidence, prevalence and mortality in the UK between 2004 and 2014. Diabetes, Obesity & Metabolism.

[30] Rowen D, Brazier J, Glied S, Smith P (2011). Health Utility Measurement. The Oxford Handbook of Health Economics.

[31] Rabin R, Gudex C, Selai C, Herdman M (2014). From translation to version management: a history and review of methods for the cultural adaptation of the EuroQol five-dimensional questionnaire. Value in Health.

[32] van Hout B, Janssen MF, Feng YS, Kohlmann T, Busschbach J, Golicki D (2012). Interim scoring for the EQ-5D-5L: mapping the EQ-5D-5L to EQ-5D-3L value sets. Value in Health.

[33] Janssen MF, Lubetkin EI, Sekhobo JP, Pickard AS (2011). The use of the EQ-5D preference-based health status measure in adults with Type 2 diabetes mellitus. Diabetic Medicine.

[34] Bogelund M, Vilsboll T, Faber J, Henriksen JE, Gjesing RP, Lammert M (2011). Patient preferences for diabetes management among people with type 2 diabetes in Denmark - a discrete choice experiment. Current Medical Research and Opinion.

[35] Casciano R, Malangone E, Ramachandran A, Gagliardino JJ (2011). A quantitative assessment of patient barriers to insulin. International Journal of Clinical Practice.

[36] de Bekker-Grob EW, Essink-Bot ML, Meerding WJ, Koes BW, Steyerberg EW (2009). Preferences of GPs and patients for preventive osteoporosis drug treatment: a discrete-choice experiment. PharmacoEconomics.

[37] de Bekker-Grob EW, Essink-Bot ML, Meerding WJ, Pols HA, Koes BW, Steyerberg EW (2008). Patients' preferences for osteoporosis drug treatment: a discrete choice experiment. Osteoporosis International : A Journal Established as Result of Cooperation Between the European Foundation for Osteoporosis and the National Osteoporosis Foundation of the USA.

[38] Doyle S, Lloyd A, Birt J, Curtis B, Ali S, Godbey K (2012). Willingness to pay for obesity pharmacotherapy. Obesity.

[39] Jendle J, Torffvit O, Ridderstrale M, Ericsson A, Nilsen B, Bogelund M (2012). Willingness to pay for diabetes drug therapy in type 2 diabetes patients: based on LEAD clinical programme results. Journal of Medical Economics.

[40] Jendle J, Torffvit O, Ridderstrale M, Lammert M, Ericsson A, Bogelund M (2010). Willingness to pay for health improvements associated with anti-diabetes treatments for people with type 2 diabetes. Current Medical Research and Opinion.

[41] Wilke T (2009). Patient preferences for an oral anticoagulant after major orthopedic surgery: results of a german survey. The Patient.

[42] Huang ES, Shook M, Jin L, Chin MH, Meltzer DO (2006). The impact of patient preferences on the cost-effectiveness of intensive glucose control in older patients with new-onset diabetes. Diabetes Care.

[43] Matza LS, Deger KA, Vo P, Maniyar F, Goadsby PJ (2019). Health state utilities associated with attributes of migraine preventive treatments based on patient and general population preferences. Quality of Life Research.

[44] Ara R, Brazier J (2017). Estimating Health State Utility Values for Comorbidities. PharmacoEconomics.

[45] Ara R, Wailoo A (2011). NICE DSU Technical Support Document 12: The Use of Health State Utility Values in Decision Models.

[46] Matza LS, Sapra SJ, Dillon JF, Kalsekar A, Davies EW, Devine MK (2015). Health state utilities associated with attributes of treatments for hepatitis C. The European Journal of Health Economics.

[47] Hochberg Y, Benjamini Y (1990). More powerful procedures for multiple significance testing. Statistics in Medicine.

