# Supplementary material for: Health state utilities associated with treatment process for oral and injectable GLP-1 receptor agonists for type 2 diabetes
Source: Qual Life Res. 2021 Apr 22;30(7):2033–43. doi: 10.1007/s11136-021-02808-2 (PMC8233232; doi:10.1007/s11136-021-02808-2)
Supplement: Supplementary file 1 — Supplementary file1 (DOCX 17 KB) [file 11136_2021_2808_MOESM1_ESM.docx]

# APPENDIX A: TEXT OF THE HEALTH STATE VIGNETTES

**Health State A: Simple Oral Treatment Process**

**Type 2 diabetes**

- You have had type 2 diabetes for several years.
- You are at your **current weight.**
- Your blood sugar levels are **usually** **in control,** but **sometimes** your blood sugar is too high or too low.
  - If your blood sugar level is **too low**, you may experience dizziness/light-headedness, sweating, or shaking.
  - If your blood sugar level is **too high**, you may experience tiredness, blurred vision, thirst, or frequent urination.

**Oral medication**

- You take an oral medication (tablet) **every day**.

**Health State B: Oral Semaglutide Treatment Process**

**Type 2 diabetes**

- You have had type 2 diabetes for several years.
- You are at your **current weight.**
- Your blood sugar levels are **usually** **in control,** but **sometimes** your blood sugar is too high or too low.
  - If your blood sugar level is **too low**, you may experience dizziness/light-headedness, sweating, or shaking.
  - If your blood sugar level is **too high**, you may experience tiredness, blurred vision, thirst, or frequent urination.

**Oral medication with requirements**

- You take an oral medication (tablet) **every day** with the following requirements:
- Take this tablet on an **empty stomach when you first wake up**.
- Take this tablet **with a sip of plain water (no more than 4 ounces)**.
- Do not split, crush, or chew tablets. **Swallow the tablets whole**.
- **Wait at least 30 minutes** after taking this tablet before eating, drinking, or taking other oral medications.
- The medication **works best if you eat 30 to 60 minutes after taking it**.

**Health State C: Dulaglutide Treatment Process**

**Type 2 diabetes**

- You have had type 2 diabetes for several years.
- You are at your **current weight.**
- Your blood sugar levels are **usually** **in control,** but **sometimes** your blood sugar is too high or too low.
  - If your blood sugar level is **too low**, you may experience dizziness/light-headedness, sweating, or shaking.
  - If your blood sugar level is **too high**, you may experience tiredness, blurred vision, thirst, or frequent urination.

**Treatment injection**

- You give yourself an injection **once each week** using a device called a pre-filled pen. The pen looks like this.
- **Each device is used once.**  You use a new pen each week.
- **Needle:**
- When you open the pen package, **the needle is already included** as a part of the injection pen. You **do NOT need to handle the needle** or attach it to the pen.
- The needle is **not visible** while you are preparing or using the device.
- When you press the injection button, the pen will automatically insert the needle into your skin, inject the medicine, and pull back (retract) the needle after the injection is complete.
- **Dosing**: The device gives only one dose. **You do not need to adjust the dose.**

**How to use the injection device**

- Pull off and discard base cap.
- Place base flat and firmly against your skin at the injection site.
- Unlock by turning the lock ring.
- Press and hold the injection button. You will hear a loud click.
- Continue holding the base firmly against your skin until you hear a second click. This occurs when the needle automatically starts retracting in about 5 to 10 seconds.
- You will know your injection is complete when a grey part becomes visible.
- Remove the pen from your skin.

**Health State D: Injectable Semaglutide Treatment Process**

**Type 2 diabetes**

- You have had type 2 diabetes for several years.
- You are at your **current weight.**
- Your blood sugar levels are **usually** **in control,** but **sometimes** your blood sugar is too high or too low.
  - If your blood sugar level is **too low**, you may experience dizziness/light-headedness, sweating, or shaking.
  - If your blood sugar level is **too high**, you may experience tiredness, blurred vision, thirst, or frequent urination.

**Injection treatment**

- You give yourself an injection **once each week** using a device called a pre-filled pen. The pen looks like this.
- **Each device is used multiple times**. Always use a new needle for each injection.
- **Needle:**
- You have to **handle the needle** and attach it to the pen.
- The needle is **visible** while you are preparing and using the device.
- After the medication has been injected, you have to remove the needle from your skin. The needle **will not retract automatically**.
- **Dosing:** You **must select the dose** before administering the injection.

**How to use the injection device**

- Pull off the pen cap.
- Take a new needle and tear off the paper tab.
- Push the needle straight onto the pen. Turn until it is on tight.
- Pull off outer cap of the needle. Pull off inner cap of the needle.
- Turn the dose selector until the dose counter stops and shows your dose. In this example, your dose is 0.5 mg.
- If the dose counter stops before your dose number, that means the pen does not have enough medication left for a full dose. In that case, you would need to discard the pen and start using a new pen.
- Insert the needle into your skin.
- Make sure you can see the dose counter. Do not cover the dose counter with your fingers. This could interrupt the injection.
- Press and hold down the dose button until the dose counter shows 0. You may hear or feel a click.
- After the dose counter has returned to 0, count slowly to 6. Do not remove the needle before counting to 6. This is to make sure that you get your full dose.
- Remove the needle from your skin.

**How to remove the needle from the pen**

- Lead the needle tip into the outer needle cap on a flat surface without touching the needle or the outer needle cap.
- Once the needle is covered, carefully push the outer needle cap completely on.
- Unscrew the needle and dispose of it.
- Put the cap on the pen.
